# The impact of maternal emotional intelligence and parenting style on child anxiety and behavior in the dental setting

**DOI:** 10.4317/medoral.17839

**Published:** 2012-08-28

**Authors:** Naser-Asl Aminabadi, Maryam Pourkazemi, Jalil Babapour, Sina-Ghertasi Oskouei

**Affiliations:** 1 Associate professor, Department of Pediatric Dentistry, Faculty of Dentistry, Tabriz University of Medical Sciences, Tabriz, Iran; 2 Postgraduate student, Department of Pediatric Dentistry, Faculty of Dentistry, Tabriz University of Medical Sciences, Tabriz, Iran; 3 Assistant professor, Department of Psychology, Tabriz University, Tabriz, Iran; 4 Research assistant, Faculty of Dentistry, Tabriz University of Medical Sciences, Tabriz, Iran

## Abstract

Objective. The present study investigated the correlations between maternal emotional intelligence (EQ), parenting style, child trait anxiety and child behavior in the dental setting. Study design. One-hundred seventeen children, aged 4-6 years old (mean 5.24 years), and their mothers participated in the study. The BarOn Emotional Quotient Inventory and Bumrind�s parenting style questionnaire were used to quantify maternal emotional intelligence and parenting style. Children�s anxiety and behavior was evaluated using the Spence Children�s Anxiety Scale (SCAS) and Frankl behavior scale. Results. Significant correlation was found between maternal EQ and child behavior (r=0.330; p<0.01); but not between parenting style and child behavior. There was no significant correlation between mother�s total EQ and child�s total anxiety; however, some subscales of EQ and anxiety showed significant correlations. There were significant correlations between authoritarian parenting style and separation anxiety (r=0.186; p<0.05) as well as authoritative parenting style and mother�s EQ (r=0.286; p<0.01). There was no significant correlation between child anxiety and behavior (r = -0.81). Regression analysis revealed maternal EQ is effective in predicting child behavior (?=0.340; p<0.01). Conclusion. This study provides preliminary evidence that the child�s behavior in the dental setting is correlated to mother�s emotional intelligence. Emotionally intelligent mothers were found to have predominantly authoritative parenting style.

** Key words:**Anxiety, child behavior, parenting, pediatric dentistry.

## Introduction

The patient-practitioner interrelationship in adult dentistry is transformed into the more complicated patient-parent-dentist interaction in pediatric dentistry (1,2). The response of a child patient to the demands of the dental treatment is complex and determined by the impact of background variables such as personality traits and parental factors (3). The infant�s behavior will start to follow a pattern that is built up through its relationship with the mother. The attitudes and the emotions of parents have profound effects on the emotional development of children (4). Previous studies suggest that non-responsive parents have children who do not cope adaptively with stress and experience reduced interaction in social interactions (5). Likewise, parents who experience more positive effect and who share more that positive affect with their children have children who display greater emotional skills (6). By far the most influential research on parenting style has been based on Baumrind�s original conceptualizations involving three typologies of authoritative, authoritarian, and permissive (7).

An additional factor implicated in playing role in the emotional-expressive behaviors of children is parental levels of emotional intelligence (8). Emotional intelligence refers to an ability to recognize the meaning of emotions and their relationships and to reason and problem-solve on the basis of them. It has been suggested that emotional intelligence can be regarded as a coping mechanism which facilitates successful and efficient self-regulation toward desired ends (9). Studies have proposed a substantial role for parents in the development of their children�s emotional competences that provide children with the flexibility to respond to stressful life events in a resilient way (10,11). Accordingly, various aspects of parent-child interactions have been suggested as contributing to child anxiety disorders and behavior problem (12,13). It seems that emotional intelligence of mother may be one of the most important factors in development of preschool child�s anxiety and behavior in stressful conditions such as the dental setting.

Anxiety leads to increased environmental and somatic scanning that facilitates sensory receptivity. Patients with generalized anxiety are hypervigilant about their internal bodily states which should increase attention to pain (14,15). Alwin et al. (16) found no difference in general anxiety between the groups of cooperative and uncooperative patients. However, Klorman et al. (17) found that the patient�s trait anxiety was correlated with uncooperativeness in two studies of their three studies, but did not predict behavior profile scores in any sample.

The present study was designed to explore the possible correlations between maternal emotional intelligence (EQ) and parenting style with child trait anxiety and child behavior in the dental setting as a stressful condition. In addition, we assessed correlation between maternal emotional intelligence and mother�s parenting style. We hypothesized that parenting style of mother and mother�s emotional intelligence correlate with child�s behavior in the dental setting and child�s anxiety. We also hypothesized that child�s trait anxiety negatively correlates to behavior in the dental setting. The findings of current study may enhance our knowledge about predictor factors associated with child�s behavior in the dental setting and facilitate our efforts in children�s behavior management.

## Material and Methods

-Study population

The participants included 128 healthy children (61 boys and 67 girls) aged 4�6 years, enrolled in the Department of Pediatric Dentistry, Tabriz University of Medical Sciences, during the period from February to April 2010. The participants were mostly referrals from the general practitioners working in the area or children under routine medical care in the Pediatric Hospital, who are usually referred to the Department of Pediatric Dentistry for comprehensive assessments as well as routine dental treatments. Subjects were examined by a postgraduate student under the supervision of a pediatric dentist. The medical history as well as the dental history was taken and a treatment plan was established for each patient. The following criteria were considered for inclusion in the study:

� No previous experience of dental operation and/or intraoral injections.

� No history of pain secondary to pulpitis or tooth infection.

� No history of unpleasant experiences in the medical settings.

� Decayed primary molar in the lower arch without pulpal involvement.

� Mother with at least a high-school diploma.

The selected subjects were in complete physical and mental health, with no confounding medical history. Final selection of subjects was done with a consecutive method.

-Assessment instruments

Mother�s emotional intelligence: Baron Emotional Quotient Inventory (EQ-i) was used for assessment of maternal emotional intelligence. Original EQ-i contains 133 items in the form of short sentences and employs a 5-point response scale with a textual response format ranging from �very seldom or not true of me� (1) to �very often true of me or true of me� (5). The EQ-i is suitable for individuals 17 years of age and older. The individual�s responses render a total EQ score and scores on the 5 composite scales that comprise 15 subscale scores: Intrapersonal (comprising Self-Regard, Emotional Self-Awareness, Assertiveness, Independence, and Self Actualization); Interpersonal (comprising Empathy, Social Responsibility, and Interpersonal Relationship); Stress Management (comprising Stress Tolerance and Impulse Control); Adaptability (comprising Reality-Testing, Flexibility, and Problem-Solving); and General Mood (comprising Optimism and Happiness). Higher EQ score suggests that the respondent is more effective in emotional and social functioning (18,19). This questionnaire has been standardized for Iranian population previously. In our study inter rater reliability of EQ-i calculated by chronbach�s alpha was 92%.

Parenting styles: Baumrind�s parenting style scale was employed. The questionnaire comprises of 30 items including 10 items for authoritative (high control, high warmth), 10 items for authoritarian (high control, low warmth), and 10 items for permissive (low control, high warmth) parenting respectively. Parents indicate how often the stated behavior is used when interacting with their children. Response choices ranged from �Completely disagree� to �Completely agree� on a 5-point scale. A summed score was tabulated as directed for each mother on each of the three parenting styles, which means that the higher the score, the more the caregiver exhibited that particular parenting style (7). Prior to the administration of the Baumrind�s parenting style scale, translated version of the instruments to Farsi language was submitted to an expert psychologist for assessment. The translated inventory was then back-translated after which all items were checked again by the translator and same psychologist. Psychometric property of this test was calculated in a pilot study with 30 subjects. The test-retest reliability for patenting style questionnaire after a two-week interval was 0.74.

Child�s anxiety: Spence Children�s Anxiety Scale (SCAS) was used to assess child anxiety. The scale consists of 28 scored anxiety items that ask parents to report on the frequency of which an item is true for their child. Each item is rated on a 5-point scale from 0 �not at all� to 4 �very often true�. The 28 anxiety items provide an overall measure of anxiety, in addition to scores on five sub-scales each tapping a specific aspect of child anxiety, namely generalized anxiety, social anxiety, obsessive compulsive disorder, physical injury fears and separation anxiety (20). Original Farsi translations were verified by back translation performed by two independent translators. Psychometric property of this test was calculated in a pilot study with 30 subjects. The inter-rater reliability test during the pilot test of anxiety questionnaire showed 82-90% agreement in each item.

 Child�s behavior: The child�s behavior during treatment was assessed according to Frankl behavior scale. Frankl scale divides observed behavior into four categories, ranging from definitely positive to definitely negative. Rating 1: Definitely Negative. Refusal of treatment, forceful crying, fearfulness or any other overt evidence of extreme negativism. Rating 2: Negative. Reluctance to accept treatment, uncooperativeness, some evidence of negative attitude but not pronounced (sullen, withdrawn). Rating 3: Positive. Acceptance of treatment; cautious behavior at times; willingness to comply with the dentist, at times with reservation, but patient follows the dentist�s directions cooperatively. Rating 4: Definitely Positive. Good rapport with the dentist, interest in the dental procedures, laughter and enjoyment (21). In our study the Kappa value for intra-examiner agreement of data for the Frankl scale was 0.75.

-Study procedure

After preliminary selection of cases, the study procedure was explained to the parents and an informed written consent was taken. The study design which was in accordance with the Helsinki Declaration of Human Rights was approved by the Committee for Ethics in Research on Humans at Tabriz University of Medical Sciences. The test administrator explained to mothers how to complete the questionnaires. Children who met the inclusion criteria in the examination visit were introduced to dental operation in another visit. Mothers were attending the treatment course. The main operator was blind to the questionnaires. The standard inferior alveolar nerve block (1 ml 2% lidocaine, epinephrine 1/100000) was administered using distraction and counter irritation method. Cavity preparations were performed and restored using amalgam. The various strategies were used for controlling the child�s behavior based on the American Academy of Pediatric Dentistry guidelines (3). Another pediatric dentist assessed the child�s behavior during treatment course. On a random basis, a third experimenter along with the second experimenter performed the behavior evaluation to allow for the assessment of inter-examiner agreement of data.

-Statistical analysis 

Based on the results obtained from a pilot study on 10 mothers and their children, there was a r coefficient= 0.39 with an alpha=0.05 between maternal total EQ score and child behavior during dental treatment, with an assumption of the power=80% and difference of 5 units in the yielded r, the number of cases were estimated roughly at 128. This estimation was done by using the Power Sample Size software, V.1.2. Following the calculation of frequencies, mean and standard deviation, Pearson�s correlation coefficient was used for assessment of correlations between mother�s emotional intelligence, child anxiety, parenting style and child�s behavior during treatment. Multivariate regression analysis was used for assessment of predicting factors of behavior. Data were examined using the SPSS 16 software for Windows.

## Results

Totally 11 patients were excluded due to not attendance to the therapeutic sessions so 117 children (56 boys and 61 girls) with a mean age of 5.24�0.31 years old (range 4-6) along with their mothers with a mean age of 32�4.23 old (range 23-41) completed the course of the study. All mothers held a high school diploma. The mean total EQ score of the mothers was 332.39 � 41.99 (min. 200 and max. 396). The mean score of authoritative, authoritarian and permissive parenting style was 32.56�4.18, 14.95�4.61 and 18.05�4.77 respectively. The mean score of anxiety scale was 53.11�14.44 (range, 38-99). The results of behavior observation revealed that 22.27% of children had score 1 on the Frankl scale. Score 2, 3 and 4 were seen in 36.8%, 9.4%, and 31.6% of the children, respectively.

Mothers� total emotional intelligence had significantly positive correlation with the child�s behavior. Also, there was significant correlation between child�s behavior and some EQ subscales ( Table 1). There was no significant correlation between mother�s total EQ and child�s total anxiety scores. There were significant correlations between child�s generalized anxiety and maternal total EQ and significant correlation between some subscales of maternal EQ and some subscales of child anxiety ( Table 1). There was no statistically significant correlation between child behavior and total anxiety scale and anxiety subscales ( Table 1). There was no significant correlation between parenting style score and the behavior score. Pearson�s correlation analysis revealed no significant correlation between parenting style and child�s total anxiety. Among subscales of anxiety, child separation anxiety was significantly correlated to authoritarian parenting style ( Table 2). Statistically significant correlation was found between maternal EQ score and authoritative parenting style. Other parenting styles were not significantly associated with maternal EQ score ( Table 2).  Table 3 summarizes the results of the regression analysis. The results revealed that among the study variables, maternal EQ positively predicts child behavior (R�=0.16) and (?= 0.34; p < 0.01). However, neither the parenting style nor the child anxiety was a significant predictor of child�s behavior.

**Table 1:**
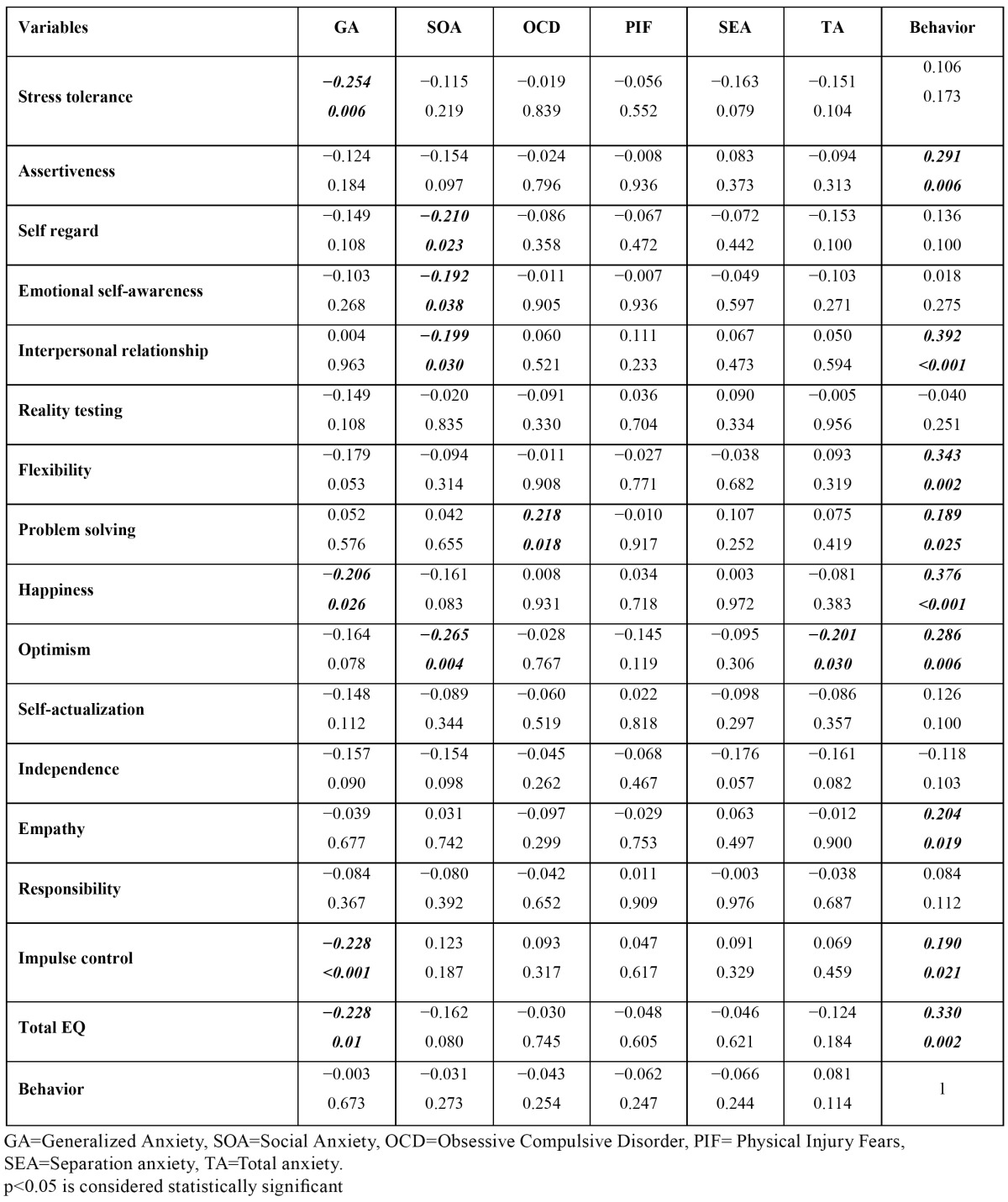
Pearson�s Correlations between maternal EQ, child� anxiety and behavior.

**Table 2:**
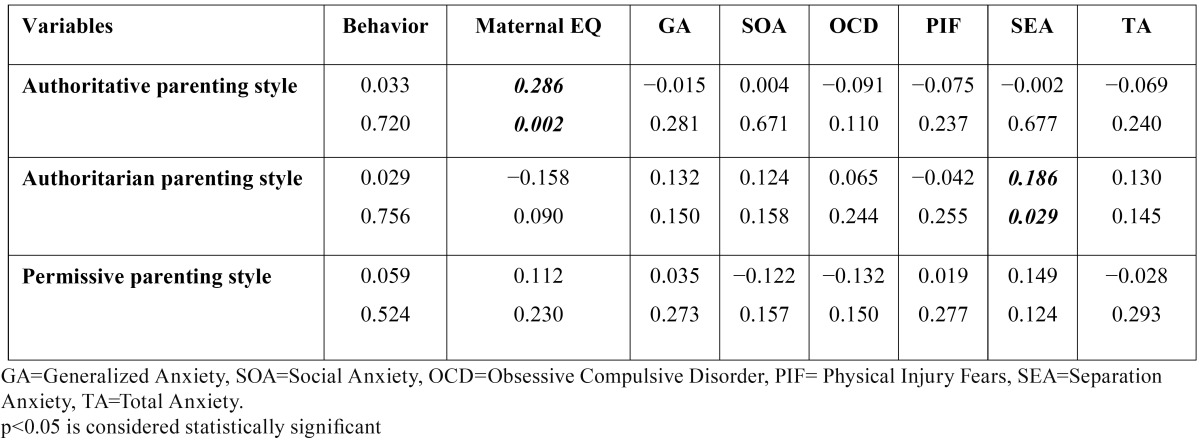
Pearson�s correlations between parenting style, child�s behavior, maternal EQ and child�s anxiety.

**Table 3:**
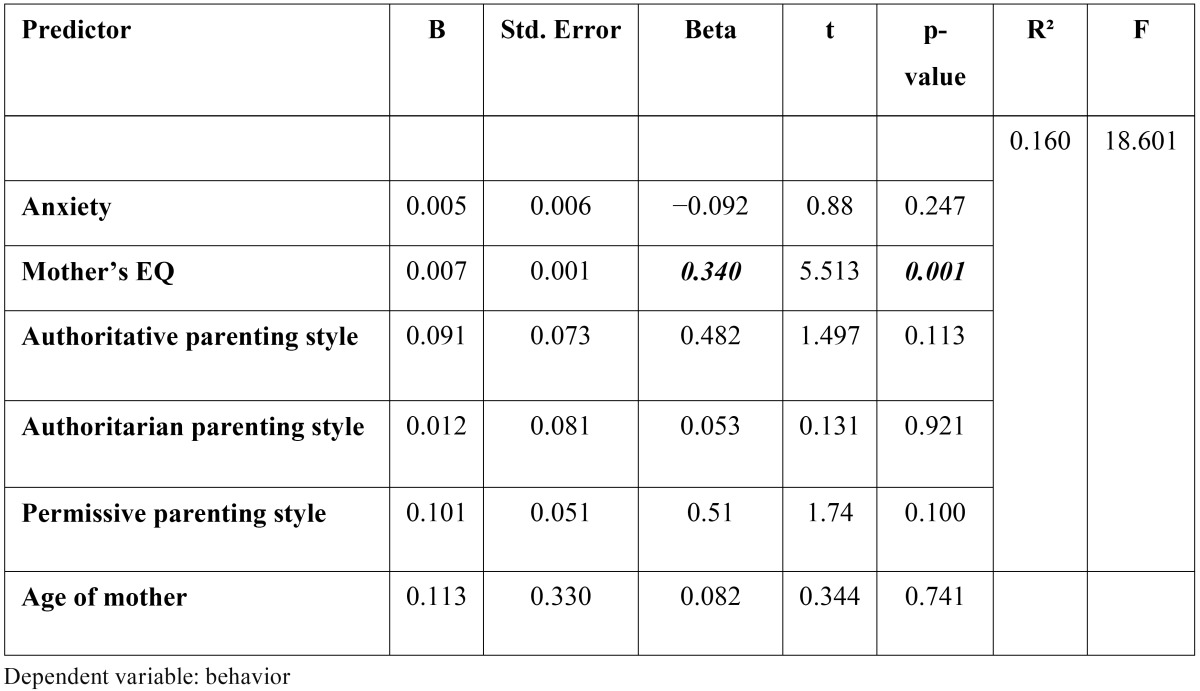
Results from multivariate regression analysis predicting behavior from maternal EQ, child anxiety and mother�s parental style.

## Discussion

The present paper provides preliminary evidence that maternal emotional intelligence positively correlates to the child�s behavior in the dental setting and can be a predictor of child�s behavior. This means children who have more emotionally intelligent mothers, have more adaptive behavior during treatment course. The way mother�s emotional intelligence influences the child behavior in the dental setting may be influenced by the child�s ability in dealing with stressful conditions. Both children�s behavior and their ability to manage emotion may be affected by interactions with the parents. Although age, gender, temperament, and development all play a unique role in regulating children�s coping responses when faced with stress, perhaps the most influential factor is exposure to the parental model of stress responses (22).

Parents� behavior constitutes the most powerful and potentially alterable influence on the developing child. Included in this parental influence is the construct of emotional intelligence, or more specifically, how parents initiate and shape a child�s emotional knowledge base. Parents facilitate their children�s emotional competence through the model they provide about expression and regulation of emotions; their reactions to children�s emotions; their discussion and coaching about emotions with their children; and the emotional contexts they put their children in (4). Parents with a high EQ may handle their emotions when they are faced with a stressful situation in a more appropriate manner and thus their children may develop their EQ by observing and learning from such role models. Outside of modeling behavior, how a parent responds to a child�s emotions under stress also contributes to how the child will adjust to the situation. Parental attribution of the child�s behavior mediates how and what parents respond (11). Therefore, it could be suggested that parents� emotional intelligence i.e. interpersonal relationship, impulse control, problem solving, assertiveness and other component of emotional intelligence is most likely transferred to their children through daily interactions. Therefore, children of emotionally intelligent mothers are also emotionally intelligent and can handle their emotions appropriately in stressful conditions such as dental setting.

Child�s generalized anxiety was negatively correlated with maternal total EQ and EQ subscales of stress tolerance, impulse control and happiness. Moreover, social anxiety of child negatively correlated with EQ subscales of interpersonal relationship, self regard, optimism and emotional self-awareness. It can be said that mother�s low EQ and her impairment in domain of stress tolerance and impulse control manifests in child�s generalized anxiety. Although the results showed no significant relationship between parenting style and child�s total anxiety, child�s separation anxiety subscale was significantly correlated to authoritarian parenting style. One important area that has been emphasized as contributing to the development of childhood anxiety is parenting. Children who perceive their parents as warm and less controlling have been shown to report better active coping skills, whereas perceptions of authoritarian parenting styles are linked to higher levels of anxiety in children (15). Studies of anxious adults also suggest a connection between anxiety and parenting styles characterized by low levels of care or warmth and high levels of control or overprotection (23). Previous studies have shown significant associations between perceived parental psychological control and the presence of both anxiety symptoms and clinical anxiety disorders in children (24). A previous study indi-cated that children from a community sample who reported more separation anxiety also reported experiencing more harsh and negative parenting. Similarly, others reported that poor mother�child relationship quality predicted children�s fear of abandonment (25).

The result of this study revealed no significant relationship between parenting styles and child�s behavior during dental treatment. The literature on associations between parenting style and child behavior during dental treatment is sparse. Our previous study indicates that children with permissive and authoritarian parents show more negative behavior (26). One study indicated that parental rearing style was not related to child behavior during dental treatment. In addition, parents with an authoritative rearing style were more convinced that the behavior of their child could be managed by the dentist than parents with a permissive and neglectful rearing style, and parents who use a permissive rearing style were less likely to tell their children that the dentist will not hurt them compared with authoritarian parents (27).

Moreover the results of the present study demonstrate no significant relationship between child�s anxiety and behavior. Previous literature on the effect of anxiety on child�s behavior in the dental setting is controversial. The results of the present and previous studies indicate that trait anxiety is not necessarily associated with behavioral problems in the dental setting (16,17). These find-ings suggest that the nature of this association is complex. Child cognitive level (3), temperament/personality characteristics and coping strategy used by the child (28,29) may modulate the effect of child�s trait anxiety on behavior in the dental setting.

We also found significant positive correlation between maternal emotional intelligence and authoritative parenting style. This implies that authoritative mothers were also more emotionally intelligent. Mother�s emotional intelligence affects how she nurtures and interacts with her child. High emotional intelligence helps maintain a level of clarity that allows parents to respond to their children�s behavior in ways that encourage, rather than discourage, and raise cooperative children.

The results of current study have highlighted the importance of early parenting, and, of course parental emotional intelligence as a source of actions, feelings and beginning reflective emotional development of children. However, such conclusions should be carefully weighed, considering the fact that the relationship between maternal emotional intelligence and children�s behavior may be mediated through various intervening factors like social and cultural status of the mothers. Therefore, additional work is warranted in embedding maternal emotional intelligence and parenting styles within a given socioeconomic and cultural context. There are also some factors that may moderate or mediate the relation among mother-child, and children�s behavior and emotional development. For example family atmosphere such as belonging to nuclear or extended family, number of siblings, father authority, the parent�s own developmental history with her or his own parents and child personal characteristics, such as evolving temperament, all may affect the quality of parenting as the child develops. Regarding the various factors influencing the child-parent-dentist relationship, the extrapolation of the results of the present study to a broader sense and generalization of the findings necessitates further investigations.
